# The relationship between the haemoglobin concentration and the haematocrit in *Plasmodium falciparum *malaria

**DOI:** 10.1186/1475-2875-7-149

**Published:** 2008-08-02

**Authors:** Sue J Lee, Kasia Stepniewska, Nicholas Anstey, Elizabeth Ashley, Karen Barnes, Tran Quang Binh, Umberto D'Alessandro, Nicholas PJ Day, Peter J de Vries, Grant Dorsey, Jean-Paul Guthmann, Mayfong Mayxay, Paul Newton, Francois Nosten, Piero Olliaro, Lyda Osario, Loretxu Pinoges, Ric Price, Mark Rowland, Frank Smithuis, Robert Taylor, Nicholas J White

**Affiliations:** 1 Mahidol-Oxford Tropical Medicine Research Unit (MORU), Mahidol University, Faculty of Tropical Medicine, 3rd Floor, 60th Anniversary Chalermprakiat Building, 420/6 Ratchawithi Rd., Ratchathewi District, Bangkok 10400, Thailand; 2 Menzies School of Health Research and Royal Darwin Hospital, Darwin, NT, Australia; 3 Shoklo Malaria Research Unit, Mae Sot, Thailand; 4 University of Cape Town, South Africa; 5 Choray Hospital, Ho Chi Minh City, Vietnam; 6 Institute of Tropical Medicine, Antwerp, Belgium; 7 Centre for Clinical Vaccinology and Tropical Medicine, Oxford, UK; 8 Division of Infectious Diseases, Tropical Medicine & AIDS, Academic Medical Center, Amsterdam, The Netherlands; 9 University of California, San Francisco, CA, USA; 10 Institut de Recherche en Sciences de la Sante, Burkino Faso; 11 Uganda Malaria Surveillance Programme, Uganda; 12 Epicentre, France; 13 National University of Laos, Laos; 14 Wellcome Trust- Mahosot Hospital- Oxford University Tropical Medicine Research Collaboration, Laos; 15 WHO TDR, Geneva, Switzerland; 16 Centro Internatcional de Entrenamiento e Investigaciones Medicas, Colombia; 17 London School of Hygiene & Tropical Medicine, London, UK; 18 Médecins sans Frontières, The Netherlands

## Abstract

**Background:**

Malaria is a very important cause of anaemia in tropical countries. Anaemia is assessed either by measurement of the haematocrit or the haemoglobin concentration. For comparisons across studies, it is often necessary to derive one measure from the other.

**Methods:**

Data on patients with slide-confirmed uncomplicated falciparum malaria were pooled from 85 antimalarial drug trials conducted in 25 different countries, to assess the haemoglobin/haematocrit relationship at different time points in malaria. Using a linear random effects model, a conversion equation for haematocrit was derived based on 3,254 measurements from various time points (ranging from day 0 to day 63) from 1,810 patients with simultaneous measurements of both parameters. Haemoglobin was also estimated from haematocrit with the commonly used threefold conversion.

**Results:**

A good fit was obtained using Haematocrit = 5.62 + 2.60 * Haemoglobin. On average, haematocrit/3 levels were slightly higher than haemoglobin measurements with a mean difference (± SD) of -0.69 (± 1.3) for children under the age of 5 (n = 1,440 measurements from 449 patients).

**Conclusion:**

Based on this large data set, an accurate and robust conversion factor both in acute malaria and in convalescence was obtained. The commonly used threefold conversion is also valid.

## Background

Malaria is a major cause of anaemia in tropical countries. It results from the obligatory destruction of parasitized erythrocytes, the accelerated destruction of normal erythrocytes, and variable dyserythropoiesis. Anaemia is assessed either by measurement of the haematocrit or the haemoglobin concentration. Clinical and epidemiological studies of malaria use either measure, so for comparisons across studies, it is often necessary to derive one measure from the other. Malaria is associated with increased acute phase protein concentrations and severe malaria increases erythrocyte rigidity, which may affect the relationship between haemoglobin and haematocrit. Recently, it has been suggested that the threefold conversion that is commonly used to equate the two measures (haemoglobin = haematocrit/3), consistently overestimates the haemoglobin concentration resulting in an underestimate of the prevalence of anaemia [1,2].

This is important as anaemia is an important measure both of the efficacy of antimalarial treatment and the effectiveness of malaria control programmes. Data from patients with slide-confirmed, uncomplicated falciparum malaria were pooled from 85 antimalarial drug trials conducted in 25 different countries, to assess the haemoglobin/haematocrit relationship at different time points in malaria.

## Methods

There were 78,239 haematocrit (Ht) measurements from 17,739 patients compared with 26,863 haemoglobin (Hb) measurements from 13,092 patients. The conversion equation for haematocrit was based on 3,254 measurements from various timepoints from 1,810 patients with simultaneous measurements of both parameters (mean Hb 10.5 g/dl, standard deviation (SD) 2.5 g/dl; mean Ht 33.0%, SD 7.1%). Post-treatment follow-up ranged from 14 to 63 days. Haematocrit was measured by centrifugation for 673 patients and by conductivity for 1,137 patients.

Because haematocrit or haemoglobin concentrations change during recovery from malaria, adjustment for time was included. The potential effect of age and sex on the haematocrit/haemoglobin relationship was also explored.

## Results and discussion

As there was significant random variation within each individual as well as between individuals (Breusch and Pagan Lagrange multiplier test for random effects, p < 0.0001) [3] a linear random effects model was constructed using the equation:

(1) Ht_it _= α + Hb_it _* β_hb _+ time * β_time _+ v_i _+ e_it_

(where Ht is haematocrit (%), Hb is haemoglobin (g/dL), v_i _is the random effect for each individual, i, β is the coefficient for each parameter that the model estimated, e_it _is the deviation for each individual, i, at time, t, and α is the intercept) and obtained: Ht = 5.66 + 2.58 * Hb + 0.01 * time which explained approximately 80% of the variance in the model (r^2 ^(within) = 0.63, r^2 ^(between) = 0.82, r^2 ^(overall) = 0.83). Age and sex did not significantly improve the model fit (p = 0.22 and p = 0.17, respectively).

Although time contributed significantly to the model fit (95% CI 0.005, 0.023), its effect was very small. For example, a haematocrit value of 35.5% at day 0 would change to 35.78% (35.5 + 0.28) on day 28 if time were taken into account.

A simpified conversion without the time covariate using a random effects model gave:

(2) Ht = 5.62 + 2.60 * Hb

**Figure 1:**
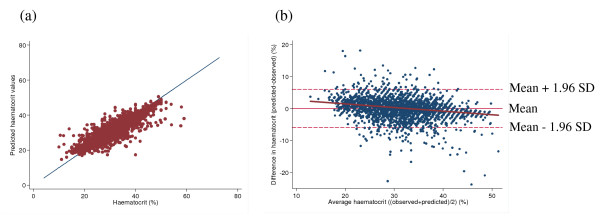
a) Plot of observed versus predicted haematocrit measurements with a line to depict the relationship with the actual haematocrit values, b) Bland-Altman plot of agreement between actual haematocrit measurements and haematocrit values estimated from haemoglobin measurements.

The model fit was still good (r^2 ^(within) = 0.63, r^2 ^(between) = 0.82, r^2 ^(overall) = 0.82; Figure 1a).

Using a Bland -Altman plot [4] to compare the values obtained using the conversion equation with the original observed values, the mean difference was -0.019 (95% CI -0.12 to 0.09) percentage points indicating that, overall, the predictions from the conversion equation tended to give slightly lower haematocrit estimates by up to 0.12 percentage points (Figure 1b). However, the conversion equation also overestimated haematocrit by up to 0.23 percentage points. Limits of agreement were calculated as the mean difference ± 1.96 SD and 94.3% of the values fell within these limits.

**Figure 2:**
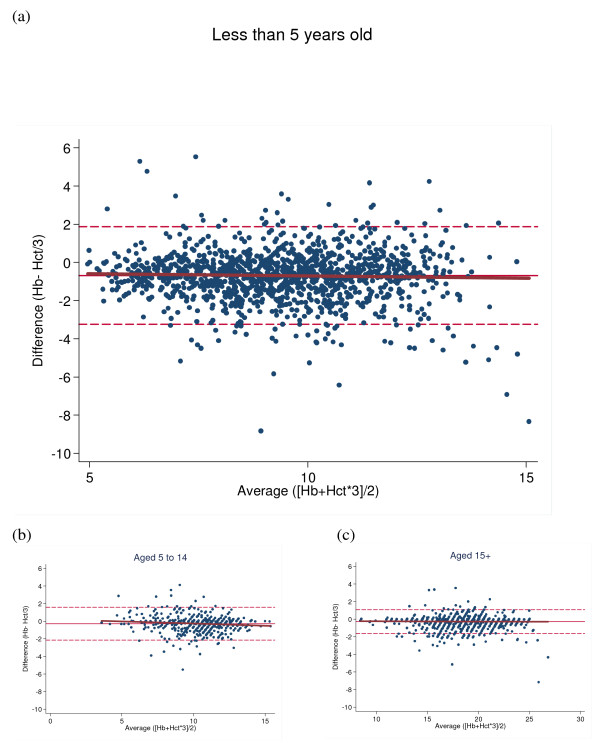
Bland-Altman plots of agreement between actual haemoglobin measurements and haemoglobin values estimated from haematocrit measurements, by age group (Note: age was missing for seven patients).

Comparing these results with those reported by Carneiro *et al*, haemoglobin was also estimated from haematocrit with the commonly used threefold conversion using data from children under the age of five only (n = 1,440 measurements from 449 patients). The Bland-Altman plot showed good agreement when comparing haemoglobin in the field and values estimated using the threefold conversion. On average, haematocrit/3 levels were slightly higher than haemoglobin measurements with a mean difference (± SD; 95% CI) of -0.69 (± 1.3; -0.75 to -0.62). 74.2% of haematocrit/3 levels were higher than haemoglobin measurements. In contrast to Carneiro *et al*, but similar to Rodriguez-Morales *et al*, larger negative differences were not observed with decreasing haemoglobin levels (Figure 2a). Agreement was also good when applying the threefold conversion to measurements from patients between five and 14 years of age (n = 501 measurements from 216 patients, Figure 2b), but was best when used in adults (15 years and older, n = 1,306 measurements from 376 patients, Figure 2c).

## Conclusion

In summary, based on this large data set, the conversion factor to equate haemoglobin and haematocrit (Ht = 5.62 + 2.60 * Hb) is accurate and robust both in acute uncomplicated malaria and in convalescence. The two measures can be readily interconverted. The commonly used threefold conversion is also valid.

## Competing interests

The authors declare that they have no competing interests.

## Authors' contributions

NJW, KS and SJL conceived the idea for paper. The data were collected from research that was designed and conducted by all the other authors. KS and SJL conducted the analysis and wrote the paper, with input from NJW and all other authors.
